# Equine Dermatophytosis: A Survey of Its Occurrence and Species Distribution among Horses in Kaduna State, Nigeria

**DOI:** 10.1155/2016/6280646

**Published:** 2016-06-01

**Authors:** Magdalene N. Maurice, Haruna M. Kazeem, Clara N. Kwanashie, Nanven A. Maurice, Emmanuel O. Ngbede, Helen N. Adamu, Wayuta P. Mshelia, Richard E. Edeh

**Affiliations:** ^1^Department of Veterinary Microbiology, Faculty of Veterinary Medicine, Ahmadu Bello University, PMB 1045, Zaria, Kaduna State, Nigeria; ^2^Department of Diagnostics and Extension, National Veterinary Research Institute, PMB 01, Vom, Plateau State, Nigeria; ^3^Department of Veterinary Pathology and Microbiology, College of Veterinary Medicine, University of Agriculture, Makurdi, PMB 2373, Benue State, Nigeria; ^4^Department of Epidemiology and Medical Statistics, Faculty of Public Health, University of Ibadan, PMB 5017, Oyo State, Nigeria; ^5^Department of Veterinary Medicine, Faculty of Veterinary Medicine, Ahmadu Bello University, PMB 1045, Zaria, Kaduna State, Nigeria

## Abstract

This study was designed to determine the occurrence and species distribution of dermatophyte from cutaneous skin lesions of horses in Kaduna State, Nigeria. A total of 102 skin scrapings were collected from 102 horses with skin lesions. Mycological studies were carried out using conventional techniques. Dermatophytes were isolated from 18 (17.6%) of the 102 samples collected. The 18 dermatophytes were distributed into 10 different species belonging to* Microsporum* (*n* = 5) and* Trichophyton* (*n* = 5) genera.* T. verrucosum* (*n* = 4) was the most predominant species isolated followed by* M. equinum* (*n* = 3),* T. vanbreuseghemii* (*n* = 2),* M. gypseum* (*n* = 2), and* M. canis* (*n* = 2). Others include* M. fulvum* (*n* = 2),* T. mentagrophytes* (*n* = 1),* T. equinum* (*n* = 1),* T. soudanense* (*n* = 1), and* M. gallinae* (*n* = 1). The present study reveals the occurrence of dermatophytes in cutaneous skin lesions of horses in Kaduna State, Nigeria. In addition for the first time in this environment the anthropophilic dermatophyte* T. soudanense* was isolated from horses. These findings have great economic, veterinary, and public health significance as they relate to the cost of treatment and dissemination of zoonotic dermatophytes.

## 1. Introduction

Dermatophytes are cited as the most frequent causes of dermatological problems in domestic animals [1]. They belong to the class Ascomycetes, which are normally located in the stratum corneum, hair shaft, or hoof, where they invade [2]. There are approximately 40 different species of dermatophytes characterized by their capability to digest keratin and divided into three genera:* Trichophyton*,* Microsporum*, and* Epidermophyton* [3]. A wide variety of dermatophytes have been isolated from animals, but a few zoophilic (*M. canis*,* T. mentagrophytes*,* T. equinum*, and* T. verrucosum*), geophilic (*M. gypseum*), and anthropophilic dermatophytes (*M. equinum* and* T. equinum*) are reported to frequently cause dermatophytosis in horses [4, 5].

The distribution of dermatomycosis, their aetiological agents, and the predominating anatomical infection patterns vary with geographical location, age of the animal, and environmental and cultural factors [6, 7]. Contagiousness among animal populations, high cost of treatment, difficulty of control measures, and the public health consequences of animal (especially horses) ringworm explain their great importance [4]. The high resistance of the dermatophyte arthroconidia in the environment, colonization of host species, and the confinement of animals in breeding areas are factors that also influence the endemicity of dermatophytosis [4].

Lesions arising from dermatophytosis have many adverse effects besides the discomfort and unsightly nuisance (esthetic) [8]. They also prevent the horses from working and interferes with their use in polo, racing, and riding because the horse will not be allowed at shows or other events (because it can transmit it to other horses), thus decreasing the cost value of the horse. Equine dermatophytosis also has considerable zoonotic importance as animals serve as reservoirs for the zoophilic dermatophytes (especially those caused by members of the* Microsporum* spp. and* Trichophyton* genera) and their infections [8, 9]. Zoophilic dermatophytes such as* T. verrucosum*,* M. canis*,* T. mentagrophytes*,* M. gypseum*, and* T. equinum* have been reported as important causes of human tinea capitis and tinea corporis in many areas of the world [10]. It has been suggested that the increasing number of reports of infections due to zoophilic dermatophytes in humans is directly linked to the persistence of this fungus in animal reservoirs [10]. Therefore knowledge of their role in cutaneous skin lesions and identification of the species may play an important role in control of outbreaks by establishing the source of infection and thereby plans to manage and control dermatophytosis.

Although dermatophytosis is worldwide in distribution, it is more prevalent in hot humid climates than in cold dry regions [6, 11]. Despite the high prevalence of dermatophytoses in Nigeria, few studies have been carried out to identify the fungal species causing cutaneous lesions in horses and their prevalence [1]. Equine dermatophytosis has received little attention in Nigeria especially in the northern part of the country where a large population of horses are located and used for festivities (traditional durbar), polo, racing, and pleasure riding [6, 12, 13]. As a result, an actual prevalence figure for tinea in horse is unknown in Kaduna State and Nigeria as a whole. There is therefore urgent need to update our knowledge of the epidemiology of ringworm infection in domestic animals. The aim of this study was to investigate the occurrence and species distribution of dermatophytes from horses with cutaneous lesions suggestive of dermatophytosis in Kaduna State, Nigeria.

## 2. Materials and Methods

### 2.1. Sample Collection

Samples from 102 horses with cutaneous lesions suggestive of dermatophytosis were collected from March to September 2014. Skin scrapings and hair samples were collected from the margins of the lesions according to the method of Elewski [14]. Whereas generalized lesions of an anatomic location were encountered, multiple (3-4) samples were collected from the different parts of the lesion and pooled together as one sample. Also the lesions were photographed with the aid of a digital camera (Samsung WB30F). Samples were placed in coloured (brown) envelops and transported as dry packet to the Diagnostic Laboratory of the Department of Veterinary Microbiology, Ahmadu Bello University, Zaria, for cultural isolation and identification of dermatophytes.

### 2.2. Culture and Isolation of Dermatophytes

Sabouraud dextrose agar (SDA) (Oxoid, UK) supplemented with chloramphenicol 40 mg/L (Fluka, UK), cycloheximide 500 mg/L (Sigma, Germany), and nicotinic acid (100 *μ*g/mL) was used for primary isolation [15]. Culture was carried out on agar plates. Another set of SDA (vitamin-free) was seeded concurrently. The scabs and hair collected were seeded on the medium and the plates incubated at room temperature for 1–4 weeks. The plates were checked for visible fungi growth every other day.

### 2.3. Identification of Isolates

Pure fungi growths of suspected dermatophytes from the SDA cultures plates were subcultured onto the potatoes dextrose agar (PDA) plates to facilitate sporulation [16] and incubated at room temperature for 1–4 weeks. The fungi were identified based on their colonial morphology on the agar plates and microscopic characteristics (after staining with lactophenol cotton blue and viewing using ×10 and ×40 magnification) with the aid of Fungal Colour Atlas [17]. Slide culture preparations were also made for isolates that were not identified from PDA culture stained slides. Hair perforation test was used as a diagnostic aid for some isolates [16]. Characteristics used for the identification of dermatophytes in the study included colony pigment, texture, growth rate, and morphological features such as macroconidia, microconidia, and nodular organs as well as nutritional characteristic such as amino acid requirement and growth in 5% salt supplemented SDA to differentiate* T. mentagrophytes* from other* Trichophyton* species [18, 19].

## 3. Results

**Figure 1:**
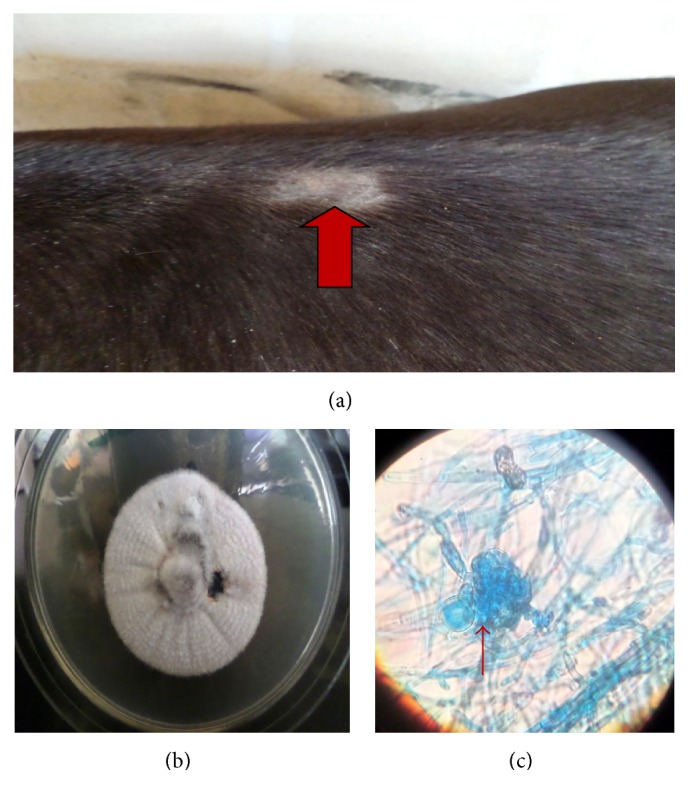
Single area of patchy alopecia in the saddle region of a horse caused by* T. equinum* (a), colony of* T. equinum* (8 days) (b), and nodular bodies and microconidia of* T. equinum* (c) (LCB ×400).

**Figure 2 fig2:**
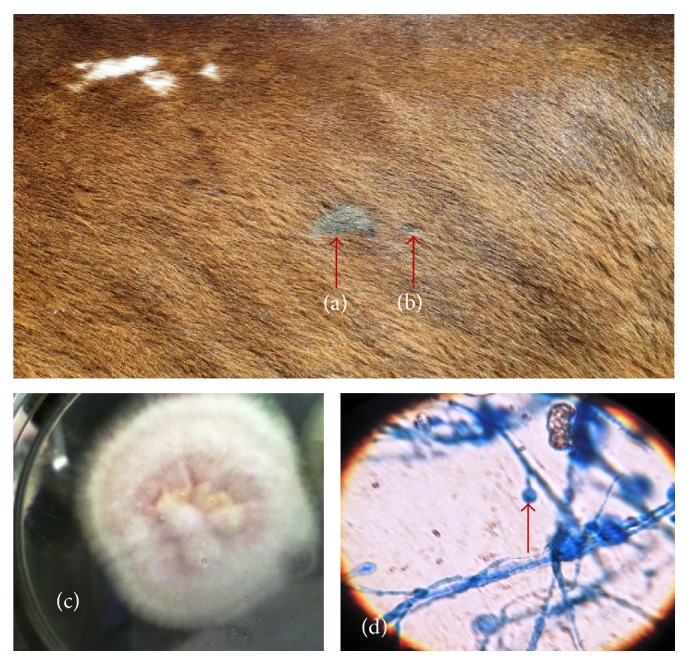
Picture showing circumscribed area of alopecia caused by* M. gallinae* on the flank of a horse (a), a satellite lesion (b), pink to cream tinged colony of* M. gallinae* (c), and pyriform microconidia of* M. gallinae* (d) (LCB ×400).

**Figure 3 fig3:**
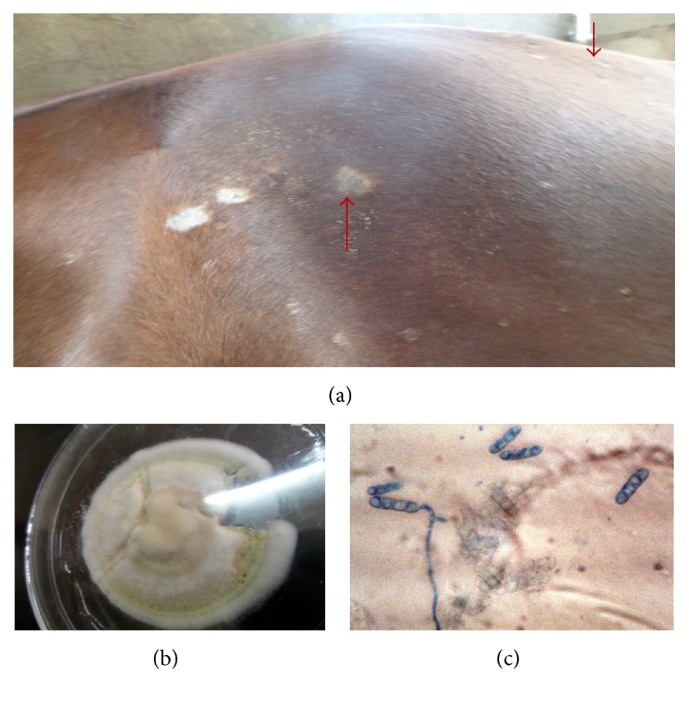
Generalized areas of alopecia on a horse, giving it a “moth eaten” appearance caused by* T. vanbreuseghemii* (a), colony of* T. vanbreuseghemii* (17 days) (b), microscopy of* T. vanbreuseghemii* (LCB ×400) showing four-celled club-shaped macroconidia, and microconidia (c).

**Figure 4 fig4:**
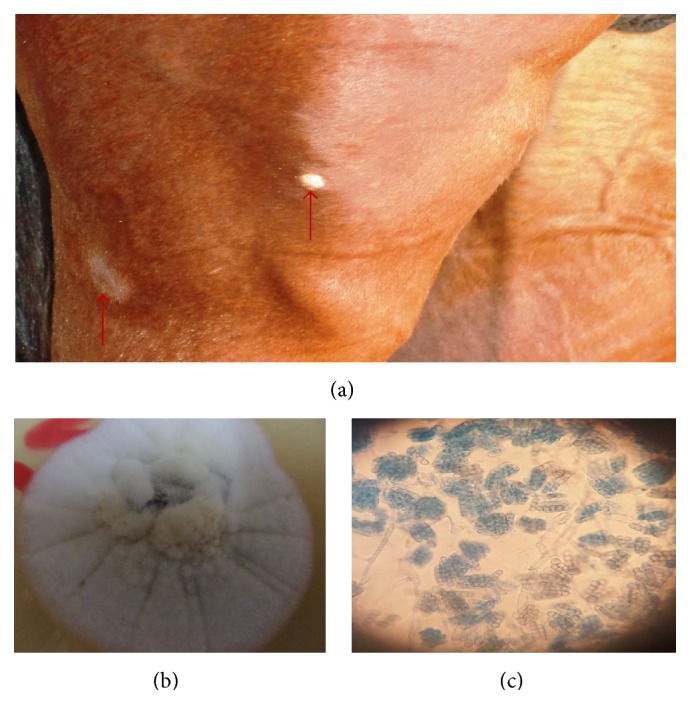
Lesions of dermatophytosis on the hind limb of a horse caused by* M. gypseum* (a), cream colony of* M. gypseum* with radial grooves and a central umbo (b), and multiple 4- celled macroconidia of* M. gypseum* in clusters (c).

**Figure 5 fig5:**
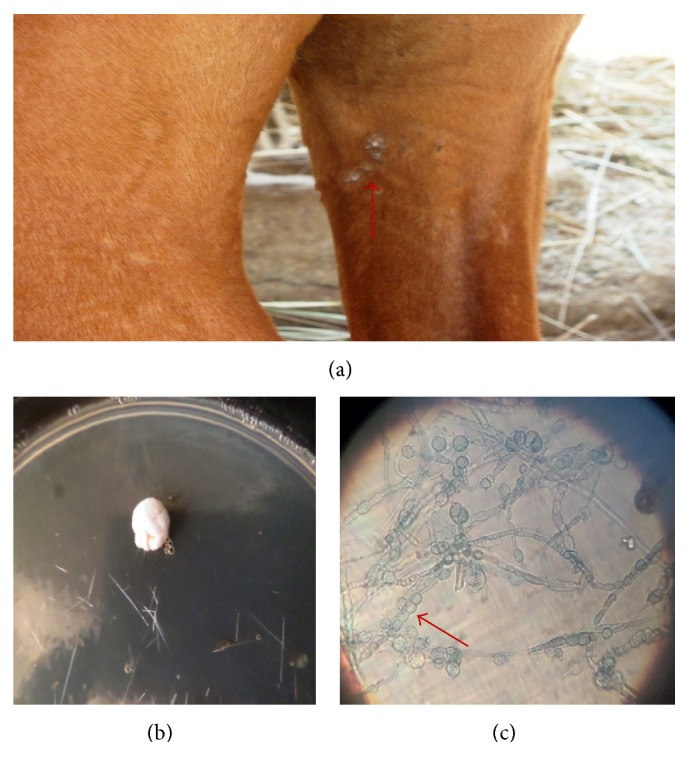
Dermatophytosis caused by* T. verrucosum* on the limb of a horse (a), slow growing button shaped colony of* T. verrucosum* (12 days) (b), and chlamydospores in chains (c) (LCB ×400).

**Figure 6 fig6:**
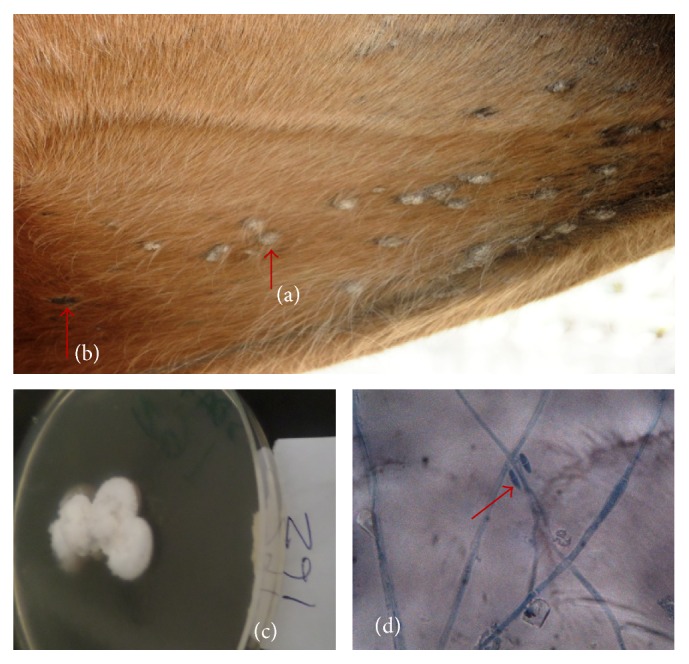
Discrete, raised areas of crusting (a), progressing to areas of patchy alopecia on the girth region of a horse caused by* M. equinum* (b), downy colony of* M. equinum* (c), and two-celled thick walled spindle shaped macroconidia of* M. equinum* (d).

**Figure 7 fig7:**
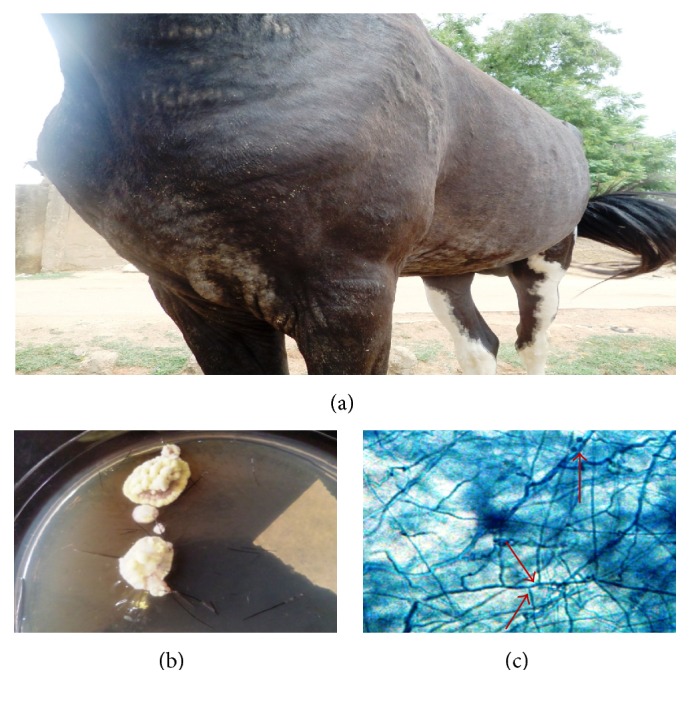
Areas of hyperkeratosis and alopecia caused by* T. sudanense* (a), yellow to apricot heaped granular colony of* T. sudanense* (b), and few globuse microconidia with right angle branching hyphae (c) (LCB ×400).

**Figure 8 fig8:**
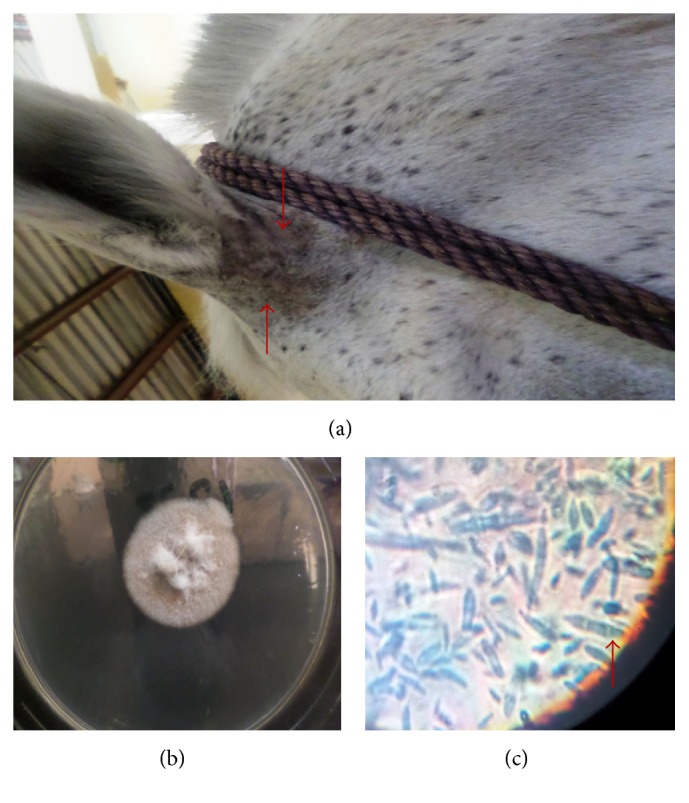
Area of alopecia on the base of an ear of a horse from which* M. fulvum* was isolated (a), pinkish-buff colony of* M. fulvum* (8 days) with central white tuft of mycelium (b), and one- to three-celled macroconidia with tapered ends (c) (LCB ×400).

**Figure 9 fig9:**
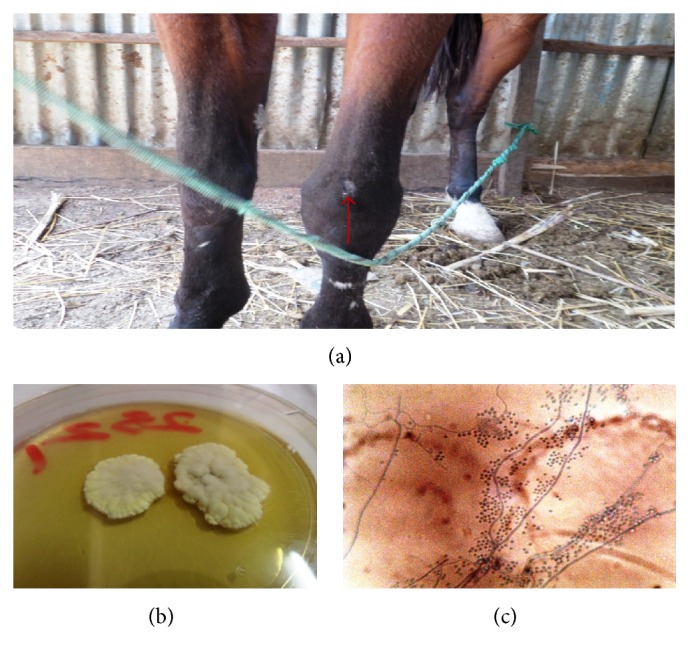
Lesion caused by* T. mentagrophytes* on the limb of a horse (a), granular colony of* T. mentagrophytes* (b), and typical microconidia of* T. mentagrophytes* “bunch of grapes” formation (c) (LCB ×400).

**Figure 10:**
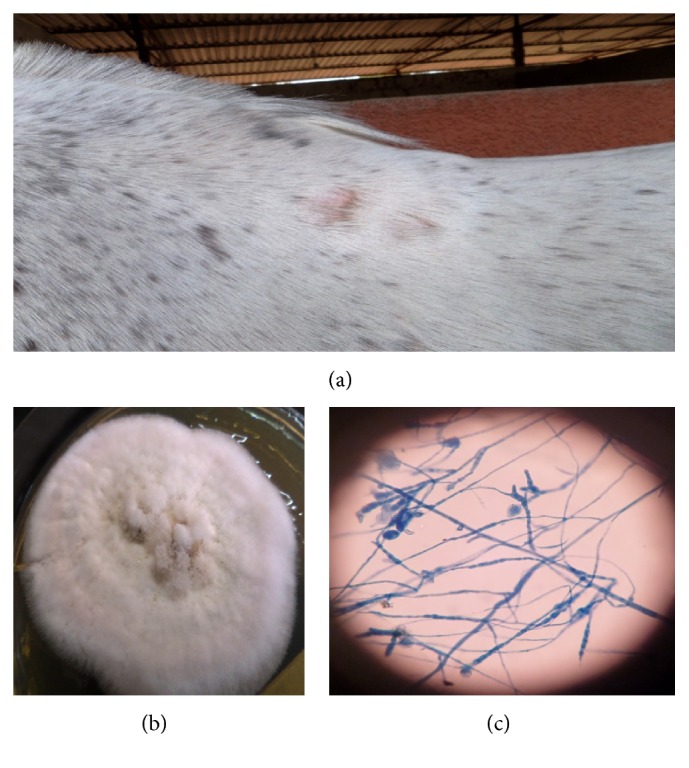
Multiple areas of alopecia on the back of a horse caused by* M. canis* (a), colony of* M. canis* (17 days) (b), and microscopy of* M. canis* showing curved spindle shaped macroconidia with a few distorted, and chlamydospores (c) (LCB ×400).

The study examined 102 horses comprising 53 males and 49 females and aged between six months to 20 years, with cutaneous skin lesion suggestive of dermatophytosis. Out of these 102 horses sampled, 18 (17.6%) of the samples were positive for dermatophytes. Majority (33.3%) of the dermatophytes were isolated from the saddle area (Table 1). The dermatophytes were distributed across two genera* Microsporum* and* Trichophyton* and 10 different species (Table 2).* Trichophyton verrucosum* was the most commonly occurring dermatophytes with a frequency of four and occurred mostly on the limbs and rump, with areas of inflammation (kerion) (Figures 1 [Fig fig2] [Fig fig3] [Fig fig4] [Fig fig5] [Fig fig6] [Fig fig7] [Fig fig8] [Fig fig9]–10). This was followed closely by* Microsporum equinum* (Figure 1) which was isolated from lesions on the saddle, flank, and girth areas (pressure areas).* T. Sudanense* (Figure 7), an anthropophilic dermatophyte, was isolated from the girth area of a horse. Lesions were found to be areas of patchy alopecia. Lesions of dermatophytosis and isolates were found on the limbs and saddle areas (5 isolates each) followed by the flanks of horses (2 isolates) while the least was on the head and rump (1 isolate each) (Table 2).

## 4. Discussion

Lesions suggestive of dermatophytosis in this study were areas of scaling, crusting, and alopecia with some kerion formation as dermatophytes are known to digest the skin, hair, and hoof of animals as a source of carbon using proteolytic and lipolytic enzymes [2]. The lesions were found to occur mostly on the limbs and pressure areas, which is in agreement with other authors [20]. The annular and coalesced lesions expanding centrifugally and losing their circular appearance observed on some horses have been reported to be the characteristic of* Trichophyton* infection in horses [21]. Dermatophytes are one of the commonest skin diseases affecting horses [22]. Species of dermatophytes belonging to the* Trichophyton* and* Microsporum* genera were the major dermatophytes detected in this study. This finding is in agreement with the reports of previous studies that dermatophytes in horses are majorly caused by members of these 2 genera [22]. Similar species of dermatophytes with the exception of* T. soudanense* detected in this study have also been isolated from horses in other parts of the world [23].

Dermatophytes from the three ecological groups were isolated in this study,* M*.* gypseum*,* M. fulvum*, and* T. vanbreuseghemii* which are geophilic dermatophytes were isolated from different parts of the body and may have infected the horses directly from the soil and through spores infested fomites as they were all housed in different stables; another source of infection could have been asymptomatic carriers in the stables.* T. verrucosum* and* M. equinum* were the most common dermatophytes detected in this study a finding that is consistent with the reports that these agents are the common cause of equine dermatophytosis [22]. The zoophilic* T. verrucosum* could have been contracted from large herds of cattle that can be found in this region that usually share pasture with the horses [1].* T. mentagrophytes* isolated from the limb of a horse could have been contracted from rodents that have free rein of the stables and* M. canis* from dogs, cats, or any other domestic animals as they have been reported to be the most common dermatophyte encountered in domestic animals in the region [1].* T. soudanense*, an anthropophilic dermatophyte which is believed to be strictly human pathogen, was encountered in this study in a horse with extensive inflammation extending from the flank to the ventrum of the horse. This could be attributed to the close contact that exists between these horses and humans. The close contact that exists during grooming, riding, and exercising of the horse may have predisposed it to infection with the anthropophilic dermatophyte. This dermatophyte has previously been isolated from prepubescent children in Nigeria by Nweze [24]. In addition other dermatophytes detected such as* M. gypseum*,* M. canis*,* T. verrucosum*,* T. equinum*, and* T. mentagrophytes* have been isolated from cases of tinea capitis and tinea corporis among humans in Nigeria indicating the zoonotic potential of the prevalent dermatophytes [25–27]. The close contact that exists between humans and these horses especially during riding, grooming, and sporting events may result in transmission of these zoophilic dermatophytes from the horses to humans.

The detection of dermatophytes in only 17.6% of the horses suggests a role of other agents and factors in the observed cutaneous skin lesions. Poor nutrition and management, infectious diseases such as helminthosis,* Staphylococcus aureus*, infection sweat rash produced by bacteria infection of the hair follicles, allergy, and pruritis produce cutaneous lesions similar to dermatophytosis and may account for some of the skin lesions observed [28].

In conclusion, dermatophytes belonging to two genera (*Microsporum* and* Trichophyton*) were isolated from 17.6% (18/102) of the horses with cutaneous skin lesions suggestive of dermatophytosis.* T. verrucosum* was the most commonly occurring dermatophytes followed by* M. equinum*.* T. soudanense* a human dermatophyte was also isolated from one of the horses a finding that suggests a potential public health concern due to the degree of close contact between man and animals in this part of the world. Hence, it is recommended that a wide scale study encompassing different species of domestic animals in different parts of the country be carried out as this will provide more information on the role of dermatophytes in the skin lesions of animals in this environment.

## Figures and Tables

**Table 1:** Occurrence and distribution of dermatophytes based on anatomical site of lesion among horses with cutaneous skin lesions in Kaduna State, Nigeria.

Anatomical site of lesion	Number of samples	Number (%) of samples positive for dermatophytes
Saddle area	15	5 (33.3)
Limbs	20	5 (25)
Generalized	13	3 (23.1)
Rump	5	1 (20)
Flank and girth	25	3 (12)
Head	12	1 (8.3)
Neck	7	0 (0)
Wither	5	0 (0)

*Total*	*102*	*18 (17.6)*

**Table 2:** Species distribution of dermatophytes isolated from different anatomical sites of horses with cutaneous skin lesions in Kaduna State, Nigeria.

Anatomical location on the body	Total number of isolates	Species of dermatophytes isolated	Number of species of dermatophytes isolated
Head	1	*M. fulvum*	1

Limbs	5	*T. verrucosum*	3
		*M. gypseum*	1
		*T. mentagrophytes*	1

Rump	1	*T. verrucosum*	1

Saddle area	5	*M. equinum*	2
		*M. canis*	1
		*T. equinum*	1
		*M. gypseum*	1

Flank and girth	3	*M. equinum*	1
		*T. soudanense*	1
		*M. gallinae*	1

Generalized	3	*M. canis*	1
		*T. vanbreuseghemii*	2
